# Supplementary material for: Efficacy and Safety of Antibiotics in the Treatment of Methicillin-Resistant Staphylococcus aureus (MRSA) Infections: A Systematic Review and Network Meta-Analysis
Source: Antibiotics (Basel). 2024 Sep 10;13(9):866. doi: 10.3390/antibiotics13090866 (PMC11428633; doi:10.3390/antibiotics13090866)
Supplement: Supplementary file 1 [file antibiotics-13-00866-s001.zip › antibiotics-3172277-supplementary.pdf]

A systematic review and network meta-analysis of the efficacy and safety of antibiotics in the treatment of methicillin-resistant Staphylococcus aureus infections

Content list

eTable 1 PRISMA2020 checklist

eTable 2 PubMed search strategy and result

eTable 3 Embase search strategy and result

eFigure 1 Risk of bias summary

eFigure 2 Risk of bias graph

eFigure 3 Direct meta-analysis of clinical cure rates

eFigure 4 Direct meta-analysis of microbiological success rates

eFigure 5 Direct meta-analysis of the incidence of adverse events

eFigure 6 Direct meta-analysis of clinical cure rates for MRSA cSSSIs

eFigure 7 Direct meta-analysis of clinical cure rates for MRSA cSSTIs

eFigure 8 Direct meta-analysis of clinical cure rates for MRSA pneumonia

eFigure 9 Network meta-analysis of forest maps of clinical microbiology success rates

eFigure 10 Network meta-analysis of the incidence of adverse events Forest map

eFigure 11 Network meta-analysis of MRSA cSSSIs clinical cure rate Forest map

eFigure 12 Network meta-analysis of clinical cure rates for MRSA cSSTIs forest map

eFigure 13 Network meta-analysis of clinical cure rates for MRSA pneumonia Forest map

eFigure 14 SUCRA efficacy ranking curve of clinical cure success rate,SUCRA = cumulative ranking surface

eFigure 15 SUCRA efficacy ranking curve of clinical microbiology success rate, surface under SUCRA = cumulative ranking

eFigure 16 SUCRA efficacy ranking curve for the incidence of adverse reactions,SUCRA = cumulative ranking under the surface

eFigure 17 Clinical cure rate of MRSA cSSSIs SUCRA efficacy ranking curve,SUCRA = cumulative ranking under the surface

eFigure 18 Clinical cure rate of MRSA cSSTIs SUCRA efficacy ranking curve,SUCRA = cumulative ranking under the surface

eFigure 19 Clinical cure rate of MRSA pneumonia SUCRA efficacy ranking curve,SUCRA = cumulative ranking surface

eFigure 20 Funnel plot of a network meta-analysis of clinical cure success rates

eFigure 21 Funnel plot of a network meta-analysis of clinical microbiology success rates

eFigure 22 Funnel plot of a network meta-analysis of the incidence of adverse events

eFigure 23 Funnel plot of a network meta-analysis of clinical cure rates for MRSA cSSSIs

eFigure 24 Funnel plot of a network meta-analysis of clinical cure rates for MRSA cSSTIs

eFigure 25 Funnel plot of a network meta-analysis of clinical cure rates for MRSA pneumonia

Section and Topic	Item #	Checklist item	Location where item is reported
TITLE			
Title	1	Identify the report as a systematic review.	1

Section and Topic	Item #	Checklist item	Location where item is reported
ABSTRACT			
Abstract	2	See the PRISMA 2020 for Abstracts checklist.	1
INTRODUCTION			
Rationale	3	Describe the rationale for the review in the context of existing knowledge.	2
Objectives	4	Provide an explicit statement of the objective(s) or question(s) the review addresses.	2
METHODS			
Eligibility criteria	5	Specify the inclusion and exclusion criteria for the review and how studies were grouped for the syntheses.	3
Information sources	6	Specify all databases, registers, websites, organisations, reference lists and other sources searched or consulted to identify studies. Specify the date when each source was last searched or consulted.	3,6
Search strategy	7	Present the full search strategies for all databases, registers and websites, including any filters and limits used.	3
Selection process	8	Specify the methods used to decide whether a study met the inclusion criteria of the review, including how many reviewers screened each record and each report retrieved, whether they worked independently, and if applicable, details of automation tools used in the process.	3
Data collection process	9	Specify the methods used to collect data from reports, including how many reviewers collected data from each report, whether they worked independently, any processes for obtaining or confirming data from study investigators, and if applicable, details of automation tools used in the process.	3
Data items	10a	List and define all outcomes for which data were sought. Specify whether all results that were compatible with each outcome domain in each study were sought (e.g. for all measures, time points, analyses), and if not, the methods used to decide which results to collect.	5
	10b	List and define all other variables for which data were sought (e.g. participant and intervention characteristics, funding sources). Describe any assumptions made about any missing or unclear information.	5
Study risk of bias assessment	11	Specify the methods used to assess risk of bias in the included studies, including details of the tool(s) used, how many reviewers assessed each study and whether they worked independently, and if applicable, details of automation tools used in the process.	5,6
Effect measures	12	Specify for each outcome the effect measure(s) (e.g. risk ratio, mean difference) used in the synthesis or presentation of results.	6
Synthesis	13a	Describe the processes used to decide which studies were eligible for each	7

Section and Topic	Item #	Checklist item	Location where item is reported
methods		synthesis (e.g. tabulating the study intervention characteristics and comparing against the planned groups for each synthesis (item #5)).	
	13b	Describe any methods required to prepare the data for presentation or synthesis, such as handling of missing summary statistics, or data conversions.	6
	13c	Describe any methods used to tabulate or visually display results of individual studies and syntheses.	6
	13d	Describe any methods used to synthesize results and provide a rationale for the choice(s). If meta-analysis was performed, describe the model(s), method(s) to identify the presence and extent of statistical heterogeneity, and software package(s) used.	6
	13e	Describe any methods used to explore possible causes of heterogeneity among study results (e.g. subgroup analysis, meta-regression).	6
	13f	Describe any sensitivity analyses conducted to assess robustness of the synthesized results.	Not applicable
Reporting bias assessment	14	Describe any methods used to assess risk of bias due to missing results in a synthesis (arising from reporting biases).	6
Certainty assessment	15	Describe any methods used to assess certainty (or confidence) in the body of evidence for an outcome.	6
RESULTS			
Study selection	16a	Describe the results of the search and selection process, from the number of records identified in the search to the number of studies included in the review, ideally using a flow diagram.	6,17
	16b	Cite studies that might appear to meet the inclusion criteria, but which were excluded, and explain why they were excluded.	6
Study characteristics	17	Cite each included study and present its characteristics.	7
Risk of bias in studies	18	Present assessments of risk of bias for each included study.	7
Results of individual studies	19	For all outcomes, present, for each study: (a) summary statistics for each group (where appropriate) and (b) an effect estimate and its precision (e.g. confidence/credible interval), ideally using structured tables or plots.	15,16
Results of syntheses	20a	For each synthesis, briefly summarise the characteristics and risk of bias among contributing studies.	7
	20b	Present results of all statistical syntheses conducted. If meta-analysis was done, present for each the summary estimate and its precision (e.g. confidence/credible	7-10

Section and Topic	Item #	Checklist item	Location where item is reported
		interval) and measures of statistical heterogeneity. If comparing groups, describe the direction of the effect.	
	20c	Present results of all investigations of possible causes of heterogeneity among study results.	Not applicable
	20d	Present results of all sensitivity analyses conducted to assess the robustness of the synthesized results.	Not applicable
Reporting biases	21	Present assessments of risk of bias due to missing results (arising from reporting biases) for each synthesis assessed.	Not applicable
Certainty of evidence	22	Present assessments of certainty (or confidence) in the body of evidence for each outcome assessed.	7-10
DISCUSSION			
Discussion	23a	Provide a general interpretation of the results in the context of other evidence.	10
	23b	Discuss any limitations of the evidence included in the review.	11
	23c	Discuss any limitations of the review processes used.	11
	23d	Discuss implications of the results for practice, policy, and future research.	11,12
OTHER INFORMATION			
Registration and protocol	24a	Provide registration information for the review, including register name and registration number, or state that the review was not registered.	12
	24b	Indicate where the review protocol can be accessed, or state that a protocol was not prepared.	12
	24c	Describe and explain any amendments to information provided at registration or in the protocol.	Not applicable
Support	25	Describe sources of financial or non-financial support for the review, and the role of the funders or sponsors in the review.	12
Competing interests	26	Declare any competing interests of review authors.	12
Availability of data, code and other materials	27	Report which of the following are publicly available and where they can be found: template data collection forms; data extracted from included studies; data used for all analyses; analytic code; any other materials used in the review.	

From: Page MJ, McKenzie JE, Bossuyt PM, Boutron I, Hoffmann TC, Mulrow CD, et al. The PRISMA 2020 statement: an updated guideline for reporting systematic reviews. BMJ 2021;372:n71. doi: 10.1136/bmj.n71

For more information, visit: http://www.prisma-statement.org/

eTable 1 PRISMA2020 checklist

Search number	Query	Results	Time
**1**	"Methicillin-Resistant Staphylococcus aureus"[Mesh]	19,182	00:05:47
**2**	(Methicillin Resistant Staphylococcus aureus[Title/Abstract]) OR (MRSA[Title/Abstract])	36,340	00:05:55
**3**	("Methicillin-Resistant Staphylococcus aureus"[Mesh]) OR ((Methicillin Resistant Staphylococcus aureus[Title/Abstract]) OR (MRSA[Title/Abstract]))	39,761	00:06:10
**4**	"Vancomycin"[Mesh]	16,137	00:06:19
**5**	((((((((((((((((((((Vancomycin Hydrochloride[Title/Abstract]) OR (Hydrochloride, Vancomycin[Title/Abstract])) OR (Vancomycin Sulfate[Title/Abstract])) OR (Sulfate, Vancomycin[Title/Abstract])) OR (Vancomycin-ratiopharm[Title/Abstract])) OR (Vancomycin Hexal[Title/Abstract])) OR (Vancomycine Dakota[Title/Abstract])) OR (AB-Vancomycin[Title/Abstract])) OR (Vanco Azupharma[Title/Abstract])) OR (Diatracin[Title/Abstract])) OR (VANCO-cell[Title/Abstract])) OR (Vanco-saar[Title/Abstract])) OR (Vancocin[Title/Abstract])) OR (Vancocin HCl[Title/Abstract])) OR (Vancomycin Lilly[Title/Abstract])) OR (Vancocine[Title/Abstract])) OR (Vancomicina Abbott[Title/Abstract])) OR (Vancomicina Chiesi[Title/Abstract])) OR (Vancomicina Combino Phar[Title/Abstract])) OR (Vancomicina Norman[Title/Abstract])) OR (Vancomycin Phosphate (1:2[Title/Abstract]))) OR (Vancomycin Phosphate (1:2), Decahydrate[Title/Abstract])	415	00:06:28
**6**	("Vancomycin"[Mesh]) OR ((((((((((((((((((((Vancomycin Hydrochloride[Title/Abstract]) OR (Hydrochloride, Vancomycin[Title/Abstract])) OR (Vancomycin Sulfate[Title/Abstract])) OR (Sulfate, Vancomycin[Title/Abstract])) OR (Vancomycin-ratiopharm[Title/Abstract])) OR (Vancomycin Hexal[Title/Abstract])) OR (Vancomycine Dakota[Title/Abstract])) OR (AB-Vancomycin[Title/Abstract])) OR (Vanco Azupharma[Title/Abstract])) OR (Diatracin[Title/Abstract])) OR (VANCO-cell[Title/Abstract])) OR (Vanco-saar[Title/Abstract])) OR (Vancocin[Title/Abstract])) OR (Vancocin HCl[Title/Abstract])) OR (Vancomycin Lilly[Title/Abstract])) OR (Vancocine[Title/Abstract])) OR (Vancomicina Abbott[Title/Abstract])) OR (Vancomicina Chiesi[Title/Abstract])) OR (Vancomicina Combino Phar[Title/Abstract])) OR (Vancomicina Norman[Title/Abstract])) OR (Vancomycin Phosphate (1:2[Title/Abstract]))) OR (Vancomycin Phosphate (1:2), Decahydrate[Title/Abstract]))	16,260	00:07:02
**7**	"Linezolid"[Mesh]	3,555	00:07:12
**8**	(((((((((N-((3-(3-fluoro-4-morpholinylphenyl)-2-oxo-5-oxazolidinyl)methyl)acetamide[Title/Abstract]) OR (Linezolide[Title/Abstract])) OR (Zyvox[Title/Abstract])) OR (U 100766[Title/Abstract])) OR (100766, U[Title/Abstract])) OR (PNU-100766[Title/Abstract])) OR (PNU	148	00:07:19

	100766[Title/Abstract])) OR (PNU100766[Title/Abstract])) OR (U-100766[Title/Abstract])) OR (U100766[Title/Abstract])		
**9**	("Linezolid"[Mesh]) OR ((((((((((N-((3-(3-fluoro-4-morpholinylphenyl)-2-oxo-5-oxazolidinyl)methyl)acetamide[Title/Abstract]) OR (Linezolide[Title/Abstract])) OR (Zyvox[Title/Abstract])) OR (U 100766[Title/Abstract])) OR (100766, U[Title/Abstract])) OR (PNU-100766[Title/Abstract])) OR (PNU 100766[Title/Abstract])) OR (PNU100766[Title/Abstract])) OR (U-100766[Title/Abstract])) OR (U100766[Title/Abstract]))	3,617	00:07:24
**10**	"Daptomycin"[Mesh]	2,310	00:07:28
**11**	((((((Deptomycin[Title/Abstract]) OR (Cubicin[Title/Abstract])) OR (Daptomycin, 9-L beta-Aspartic Acid[Title/Abstract])) OR (Daptomycin, 9 L beta Aspartic Acid[Title/Abstract])) OR (LY-146032[Title/Abstract])) OR (LY 146032[Title/Abstract])) OR (LY146032[Title/Abstract])	165	00:07:33
**12**	("Daptomycin"[Mesh]) OR (((((((Deptomycin[Title/Abstract]) OR (Cubicin[Title/Abstract])) OR (Daptomycin, 9-L beta-Aspartic Acid[Title/Abstract])) OR (Daptomycin, 9 L beta Aspartic Acid[Title/Abstract])) OR (LY-146032[Title/Abstract])) OR (LY 146032[Title/Abstract])) OR (LY146032[Title/Abstract]))	2,335	00:07:37
**13**	"Tigecycline"[Mesh]	1,970	00:07:41
**14**	(((((TBG-MINO[Title/Abstract]) OR (9-(tert-Butylglycylamido)minocycline[Title/Abstract])) OR (Tygacil[Title/Abstract])) OR (GAR 936[Title/Abstract])) OR (GAR-936[Title/Abstract])) OR (GAR936[Title/Abstract])	80	00:07:44
**15**	("Tigecycline"[Mesh]) OR ((((((TBG-MINO[Title/Abstract]) OR (9-(tert-Butylglycylamido)minocycline[Title/Abstract])) OR (Tygacil[Title/Abstract])) OR (GAR 936[Title/Abstract])) OR (GAR-936[Title/Abstract])) OR (GAR936[Title/Abstract]))	1,983	00:07:49
**16**	"telavancin" [Supplementary Concept]	257	00:07:52
**17**	((((TD 6424[Title/Abstract]) OR (TD6424[Title/Abstract])) OR (TD-6424[Title/Abstract])) OR (Vibativ[Title/Abstract])) OR (telavancin hydrochloride[Title/Abstract])	32	00:07:56
**18**	("telavancin" [Supplementary Concept]) OR (((((TD 6424[Title/Abstract]) OR (TD6424[Title/Abstract])) OR (TD-6424[Title/Abstract])) OR (Vibativ[Title/Abstract])) OR (telavancin hydrochloride[Title/Abstract]))	271	00:07:59
**19**	"T 91825" [Supplementary Concept]	403	00:08:03
**20**	((T91825[Title/Abstract]) OR (T-91825[Title/Abstract])) OR (ceftaroline[Title/Abstract])	895	00:08:08
**21**	("T 91825" [Supplementary Concept]) OR (((T91825[Title/Abstract]) OR (T-91825[Title/Abstract])) OR (ceftaroline[Title/Abstract]))	913	00:08:12
**22**	(((((("Vancomycin"[Mesh]) OR (((((((((((((((((((((Vancomycin Hydrochloride[Title/Abstract]) OR (Hydrochloride, Vancomycin[Title/Abstract])) OR	23,379	00:08:17

	(Vancomycin Sulfate[Title/Abstract])) OR (Sulfate, Vancomycin[Title/Abstract])) OR (Vancomycin-ratiopharm[Title/Abstract])) OR (Vancomycin Hexal[Title/Abstract])) OR (Vancomycine Dakota[Title/Abstract])) OR (AB-Vancomycin[Title/Abstract])) OR (Vanco Azupharma[Title/Abstract])) OR (Diatracin[Title/Abstract])) OR (VANCO-cell[Title/Abstract])) OR (Vanco-saar[Title/Abstract])) OR (Vancocin[Title/Abstract])) OR (Vancocin HCl[Title/Abstract])) OR (Vancomycin Lilly[Title/Abstract])) OR (Vancocine[Title/Abstract])) OR (Vancomicina Abbott[Title/Abstract])) OR (Vancomicina Chiesi[Title/Abstract])) OR (Vancomicina Combino Phar[Title/Abstract])) OR (Vancomicina Norman[Title/Abstract])) OR (Vancomycin Phosphate (1:2[Title/Abstract]))) OR (Vancomycin Phosphate (1:2), Decahydrate[Title/Abstract]))) OR (("Linezolid"[Mesh]) OR (((((((((N-((3-(3-fluoro-4-morpholinylphenyl)-2-oxo-5-oxazolidinyl)methyl)acetamide[Title/Abstract]) OR (Linezolide[Title/Abstract])) OR (Zyvox[Title/Abstract])) OR (U 100766[Title/Abstract])) OR (100766, U[Title/Abstract])) OR (PNU-100766[Title/Abstract])) OR (PNU 100766[Title/Abstract])) OR (PNU100766[Title/Abstract])) OR (U-100766[Title/Abstract])) OR (U100766[Title/Abstract])))) OR (("Daptomycin"[Mesh]) OR (((((((Deptomycin[Title/Abstract]) OR (Cubicin[Title/Abstract])) OR (Daptomycin, 9-L beta-Aspartic Acid[Title/Abstract])) OR (Daptomycin, 9 L beta Aspartic Acid[Title/Abstract])) OR (LY-146032[Title/Abstract])) OR (LY 146032[Title/Abstract])) OR (LY146032[Title/Abstract])))) OR (("Tigecycline"[Mesh]) OR ((((((TBG-MINO[Title/Abstract]) OR (9-(tert-Butylglycylamido)minocycline[Title/Abstract])) OR (Tygacil[Title/Abstract])) OR (GAR 936[Title/Abstract])) OR (GAR-936[Title/Abstract])) OR (GAR936[Title/Abstract])))) OR (("telavancin" [Supplementary Concept]) OR (((((TD 6424[Title/Abstract]) OR (TD6424[Title/Abstract])) OR (TD-6424[Title/Abstract])) OR (Vibativ[Title/Abstract])) OR (telavancin hydrochloride[Title/Abstract])))) OR (("T 91825" [Supplementary Concept]) OR (((T91825[Title/Abstract]) OR (T-91825[Title/Abstract])) OR (ceftaroline[Title/Abstract])))		
**23**	(((((((((((randomized controlled trial[Title/Abstract]) OR (controlled clinical trial[Title/Abstract])) OR (random allocation[Title/Abstract])) OR (double-blind[Title/Abstract])) OR (single-blind[Title/Abstract])) OR (survival[Title/Abstract])) OR (treatment[Title/Abstract])) OR (therapy[Title/Abstract])) OR (comparison[Title/Abstract])) OR (comparative[Title/Abstract])) OR (effective[Title/Abstract])) OR (efficacy[Title/Abstract])	9,471,610	00:08:23
**24**	((("Methicillin-Resistant Staphylococcus aureus"[Mesh]) OR ((Methicillin Resistant Staphylococcus aureus[Title/Abstract]) OR (MRSA[Title/Abstract]))) AND ((((((("Vancomycin"[Mesh]) OR ((((((((((((((((((((Vancomycin Hydrochloride[Title/Abstract]) OR (Hydrochloride, Vancomycin[Title/Abstract])) OR (Vancomycin Sulfate[Title/Abstract])) OR (Sulfate, Vancomycin[Title/Abstract])) OR (Vancomycin-ratiopharm[Title/Abstract])) OR (Vancomycin Hexal[Title/Abstract])) OR (Vancomycine Dakota[Title/Abstract])) OR (AB-Vancomycin[Title/Abstract])) OR	3,229	00:08:29

	(Vanco Azupharma[Title/Abstract])) OR (Diatracin[Title/Abstract])) OR (VANCO-cell[Title/Abstract])) OR (Vanco-saar[Title/Abstract])) OR (Vancocin[Title/Abstract])) OR (Vancocin HCl[Title/Abstract])) OR (Vancomycin Lilly[Title/Abstract])) OR (Vancocine[Title/Abstract])) OR (Vancomicina Abbott[Title/Abstract])) OR (Vancomicina Chiesi[Title/Abstract])) OR (Vancomicina Combino Phar[Title/Abstract])) OR (Vancomicina Norman[Title/Abstract])) OR (Vancomycin Phosphate (1:2[Title/Abstract]))) OR (Vancomycin Phosphate (1:2), Decahydrate[Title/Abstract]))) OR (("Linezolid"[Mesh]) OR ((((((((((N-((3-(3-fluoro-4-morpholinylphenyl)-2-oxo-5-oxazolidinyl)methyl)acetamide[Title/Abstract]) OR (Linezolide[Title/Abstract])) OR (Zyvox[Title/Abstract])) OR (U 100766[Title/Abstract])) OR (100766, U[Title/Abstract])) OR (PNU-100766[Title/Abstract])) OR (PNU 100766[Title/Abstract])) OR (PNU100766[Title/Abstract])) OR (U-100766[Title/Abstract])) OR (U100766[Title/Abstract])))) OR (("Daptomycin"[Mesh]) OR (((((((Deptomycin[Title/Abstract]) OR (Cubicin[Title/Abstract])) OR (Daptomycin, 9-L beta-Aspartic Acid[Title/Abstract])) OR (Daptomycin, 9 L beta Aspartic Acid[Title/Abstract])) OR (LY-146032[Title/Abstract])) OR (LY 146032[Title/Abstract])) OR (LY146032[Title/Abstract])))) OR (("Tigecycline"[Mesh]) OR ((((((TBG-MINO[Title/Abstract]) OR (9-(tert-Butylglycylamido)minocycline[Title/Abstract])) OR (Tygacil[Title/Abstract])) OR (GAR 936[Title/Abstract])) OR (GAR-936[Title/Abstract])) OR (GAR936[Title/Abstract])))) OR (("telavancin" [Supplementary Concept]) OR (((((TD 6424[Title/Abstract]) OR (TD6424[Title/Abstract])) OR (TD-6424[Title/Abstract])) OR (Vibativ[Title/Abstract])) OR (telavancin hydrochloride[Title/Abstract])))) OR (("T 91825" [Supplementary Concept]) OR (((T91825[Title/Abstract]) OR (T-91825[Title/Abstract])) OR (ceftaroline[Title/Abstract]))))) AND ((((((((((((randomized controlled trial[Title/Abstract]) OR (controlled clinical trial[Title/Abstract])) OR (random allocation[Title/Abstract])) OR (double-blind[Title/Abstract])) OR (single-blind[Title/Abstract])) OR (survival[Title/Abstract])) OR (treatment[Title/Abstract])) OR (therapy[Title/Abstract])) OR (comparison[Title/Abstract])) OR (comparative[Title/Abstract])) OR (effective[Title/Abstract])) OR (efficacy[Title/Abstract]))		

eTable 2 PubMed search strategy and result

No.	Query	Results	Date
#1	'methicillin resistant staphylococcus aureus'/exp OR 'methicillin resistant staphylococcus aureus'	66710	13-Apr-23
#2	'methicillin-resistant staphylococcus aureus':ab,ti	31721	13-Apr-23

#3	'mrsa':ab,ti	39646	13-Apr-23
#4	#1 OR #2 OR #3	71725	13-Apr-23
#5	'vancomycin'/exp OR 'vancomycin'	121908	13-Apr-23
#6	'vancomycin hydrochloride':ab,ti	386	13-Apr-23
#7	'vancomycin hydrochloride':ab,ti	386	13-Apr-23
#8	'hydrochloride, vancomycin':ab,ti	6	13-Apr-23
#9	'vancomycin sulfate':ab,ti	2	13-Apr-23
#10	'sulfate, vancomycin':ab,ti	7	13-Apr-23
#11	'vancomycin-ratiopharm':ab,ti	0	13-Apr-23
#12	'vancomycin hexal':ab,ti	0	13-Apr-23
#13	'vancomycine dakota':ab,ti	0	13-Apr-23
#14	'ab-vancomycin':ab,ti	4	13-Apr-23
#15	'vanco azupharma':ab,ti	0	13-Apr-23
#16	'diatracina':ab,ti	0	13-Apr-23
#17	'vanco-cell':ab,ti	0	13-Apr-23
#18	'vanco-saar':ab,ti	0	13-Apr-23
#19	'vancocin':ab,ti	39	13-Apr-23
#20	'vancocin hcl':ab,ti	2	13-Apr-23
#21	'vancomycin lilly':ab,ti	2	13-Apr-23
#22	'vancocine':ab,ti	3	13-Apr-23
#23	'vancomicina abbott':ab,ti	0	13-Apr-23
#24	'vancomicina chiesi':ab,ti	0	13-Apr-23
#25	'vancomicina combino phar':ab,ti	0	13-Apr-23
#26	'vancomicina norman':ab,ti	0	13-Apr-23
#27	'vancomycin phosphate (1:2)':ab,ti	0	13-Apr-23
#28	'vancomycin phosphate (1:2), decahydrate':ab,ti	0	13-Apr-23
#29	#5 OR #6 OR #7 OR #8 OR #9 OR #10 OR #11 OR #12 OR #13 OR #14 OR #15 OR #16 OR #17 OR #18 OR #19 OR #20 OR #21 OR #22 OR #23 OR #24 OR #25 OR #26 OR #27 OR #28	121913	13-Apr-23
#30	'linezolid'/exp	26941	13-Apr-23
#31	'n-((3-(3-fluoro-4-morpholinylphenyl)-2-oxo-5-oxazolidinyl)methyl)acetamide':ab,ti	1	13-Apr-23
#32	'linezolide':ab,ti	118	13-Apr-23
#33	'zyvox':ab,ti	79	13-Apr-23
#34	'u 100766':ab,ti	20	13-Apr-23
#35	'100766, u':ab,ti	0	13-Apr-23
#36	'pnu-100766':ab,ti	15	13-Apr-23
#37	'pnu 100766':ab,ti	15	13-Apr-23
#38	'pnu100766':ab,ti	14	13-Apr-23
#39	'u-100766':ab,ti	20	13-Apr-23
#40	'u100766':ab,ti	22	13-Apr-23
#41	#30 OR #31 OR #32 OR #33 OR #34 OR #35 OR #36 OR #37 OR #38 OR #39 OR #40	26998	13-Apr-23

#42	'daptomycin'/exp OR 'daptomycin'	11722	13-Apr-23
#43	'deptomycin':ab,ti	4	13-Apr-23
#44	'cubicin':ab,ti	115	13-Apr-23
#45	'daptomycin, 9-l beta-aspartic acid':ab,ti	0	13-Apr-23
#46	'daptomycin, 9 l beta aspartic acid':ab,ti	0	13-Apr-23
#47	'ly-146032':ab,ti	27	13-Apr-23
#48	'ly 146032':ab,ti	27	13-Apr-23
#49	'ly146032':ab,ti	67	13-Apr-23
#50	#42 OR #43 OR #44 OR #45 OR #46 OR #47 OR #48 OR #49	11727	13-Apr-23
#51	'tigecycline'/exp OR 'tigecycline'	15128	13-Apr-23
#52	'tbg-mino':ab,ti	2	13-Apr-23
#53	'9-(tert-butylglycylamido)minocycline':ab,ti	0	13-Apr-23
#54	'tygacil':ab,ti	40	13-Apr-23
#55	'gar 936':ab,ti	57	13-Apr-23
#56	'gar-936':ab,ti	57	13-Apr-23
#57	'gar936':ab,ti	58	13-Apr-23
#58	#51 OR #52 OR #53 OR #54 OR #55 OR #56 OR #57	15129	13-Apr-23
#59	'telavancin'/exp OR 'telavancin'	1343	13-Apr-23
#60	'td 6424':ab,ti	13	13-Apr-23
#61	'td6424':ab,ti	13	13-Apr-23
#62	'td-6424':ab,ti	13	13-Apr-23
#63	'vibativ':ab,ti	9	13-Apr-23
#64	'telavancin hydrochloride':ab,ti	13	13-Apr-23
#65	#59 OR #60 OR #61 OR #62 OR #63 OR #64	1343	13-Apr-23
#66	'ceftaroline'/exp OR 'ceftaroline'	2549	13-Apr-23
#67	't 91825':ab,ti	9	13-Apr-23
#68	't-91825':ab,ti	9	13-Apr-23
#69	#66 OR #67 OR #68	2550	13-Apr-23
#70	#29 OR #41 OR #50 OR #58 OR #65 OR #69	145767	13-Apr-23
#71	'randomized controlled trial'/exp OR 'randomized controlled trial'	1026241	13-Apr-23
#72	'controlled clinical trial':ab,ti	24182	13-Apr-23
#73	'random allocationl':ab,ti	0	13-Apr-23
#74	'double-blind':ab,ti	221087	13-Apr-23
#75	'single-blind':ab,ti	21919	13-Apr-23
#76	'survival':ab,ti	1709469	13-Apr-23
#77	'treatment':ab,ti	7219478	13-Apr-23
#78	'therapy':ab,ti	3178133	13-Apr-23
#79	'comparison':ab,ti	1573043	13-Apr-23
#80	'comparative':ab,ti	505634	13-Apr-23
#81	'effective':ab,ti	2323160	13-Apr-23
#82	'efficacy':ab,ti	1483399	13-Apr-23

#83	#71 OR #72 OR #73 OR #74 OR #75 OR #76 OR #77 OR #78 OR #79 OR #80 OR #81 OR #82	12927620	13-Apr-23
#84	#4 AND #70 AND #83	15169	13-Apr-23

eTable 3 Embase search strategy and result

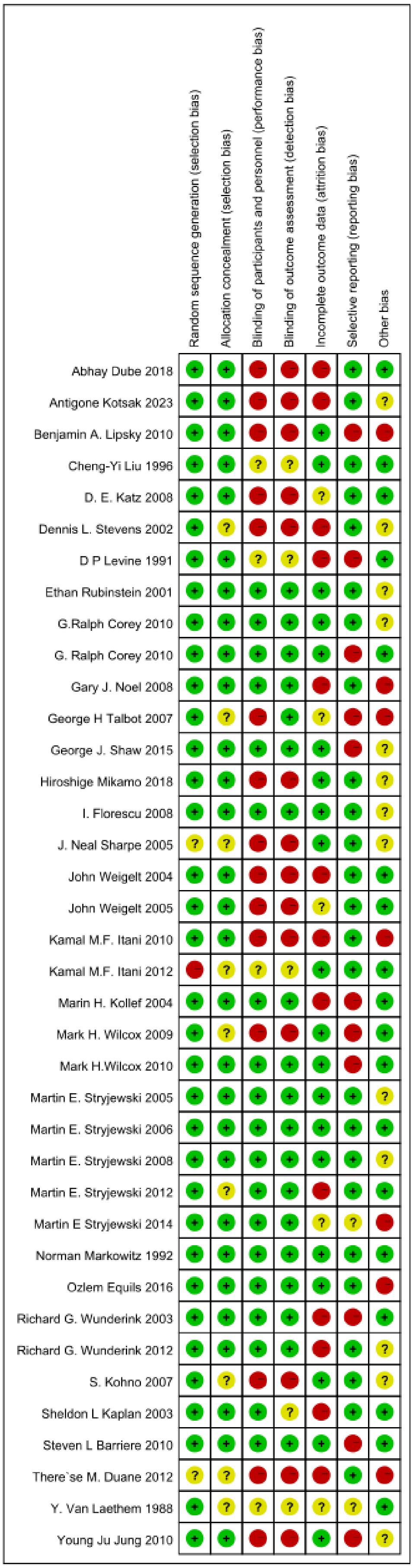

Random sequence generation (selection bias)
Allocation concealment (selection bias)
Blinding of participants and personnel (performance bias)
Blinding of outcome assessment (detection bias)
Incomplete outcome data (attrition bias)
Selective reporting (reporting bias)
Other bias
Abhay Dube 2018
Antigone Kotsak 2023
Benjamin A. Lipsky 2010
Cheng-Yi Liu 1996
D. E. Katz 2008
Dennis L. Stevens 2002
D P Levine 1991
Ethan Rubinstein 2001
G.Ralph Corey 2010
G. Ralph Corey 2010
Gary J. Noel 2008
George H Talbot 2007
George J. Shaw 2015
Hiroshige Mikamo 2018
I. Florescu 2008
J. Neal Sharpe 2005
John Weigelt 2004
John Weigelt 2005
Kamal M.F. Itani 2010
Kamal M.F. Itani 2012
Marin H. Kollef 2004
Mark H. Wilcox 2009
Mark H.Wilcox 2010
Martin E. Stryjewski 2005
Martin E. Stryjewski 2006
Martin E. Stryjewski 2008
Martin E. Stryjewski 2012
Martin E Stryjewski 2014
Norman Markowitz 1992
Ozlem Equils 2016
Richard G. Wunderink 2003
Richard G. Wunderink 2012
S. Kohno 2007
Sheldon L Kaplan 2003
Steven L Barriere 2010
There`se M. Duane 2012
Y. Van Laethem 1988
Young Ju Jung 2010

eFigure 1 Risk of bias summary

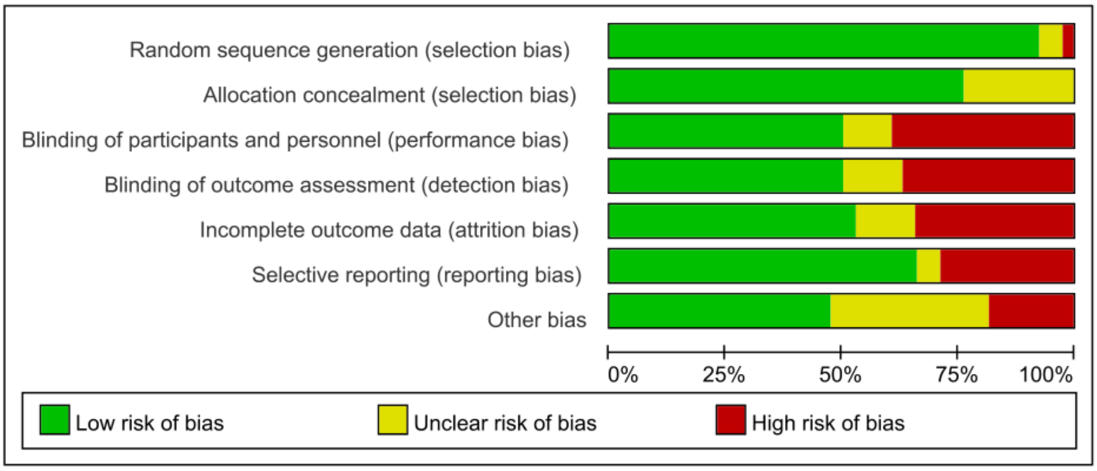

eFigure 2 Risk of bias graph

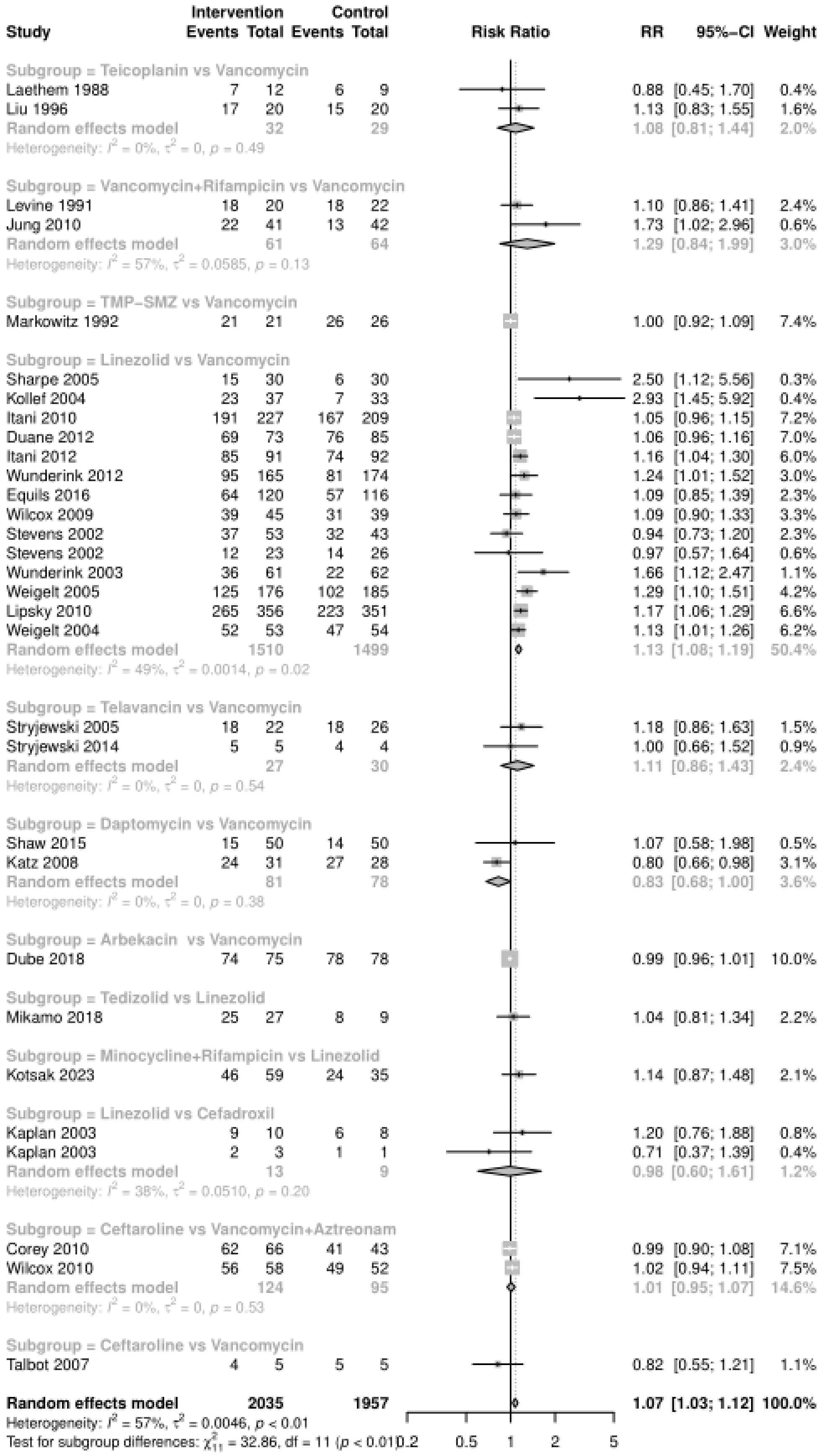

eFigure 3 Direct meta-analysis of clinical cure rates

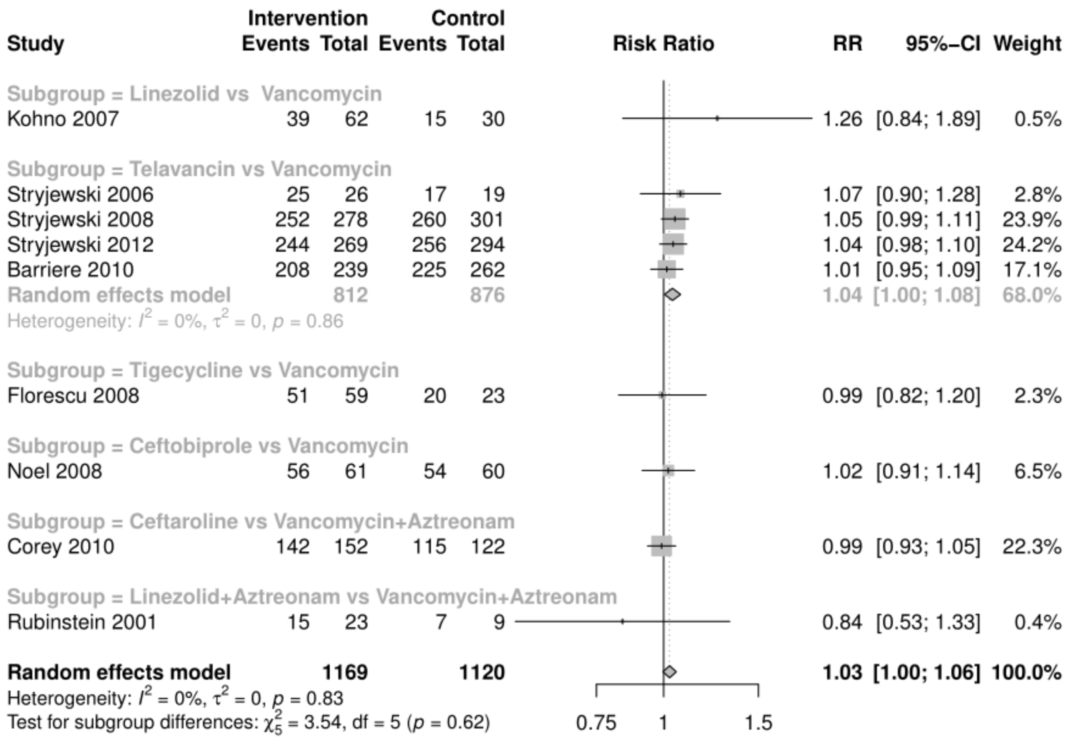

eFigure 4 Direct meta-analysis of microbiological success rates

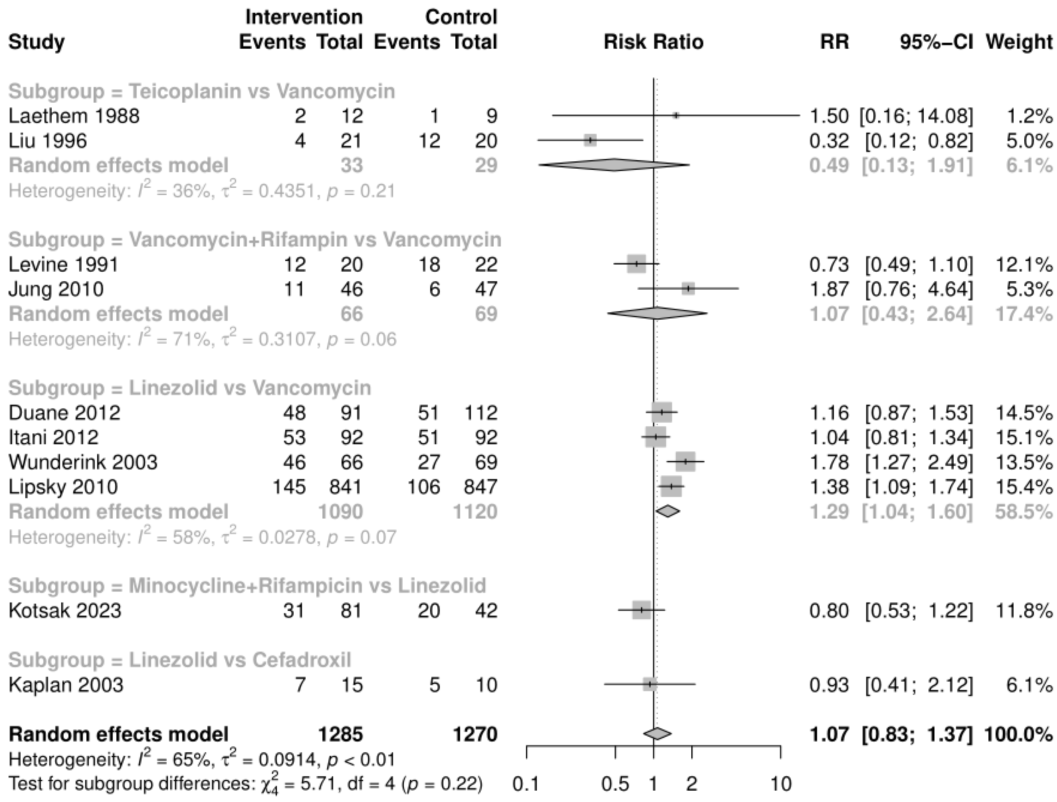

eFigure 5 Direct meta-analysis of the incidence of adverse events

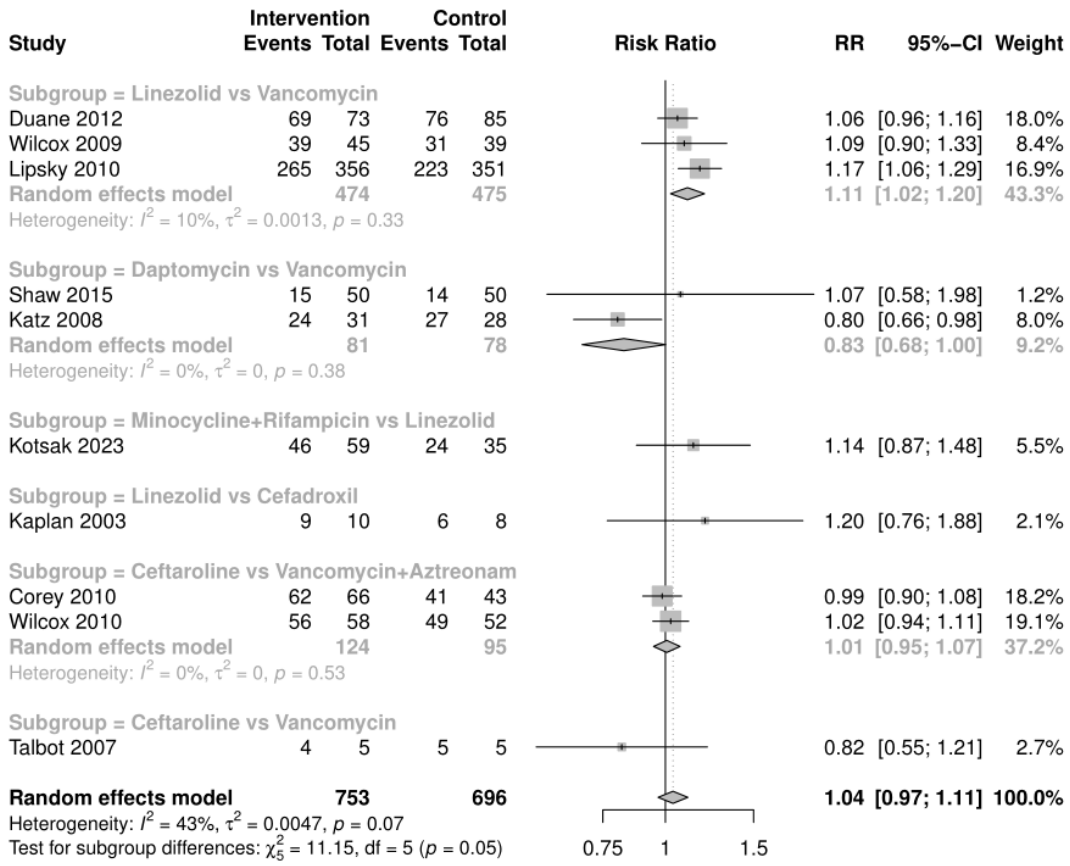

eFigure 6 Direct meta-analysis of clinical cure rates for MRSA cSSSIs

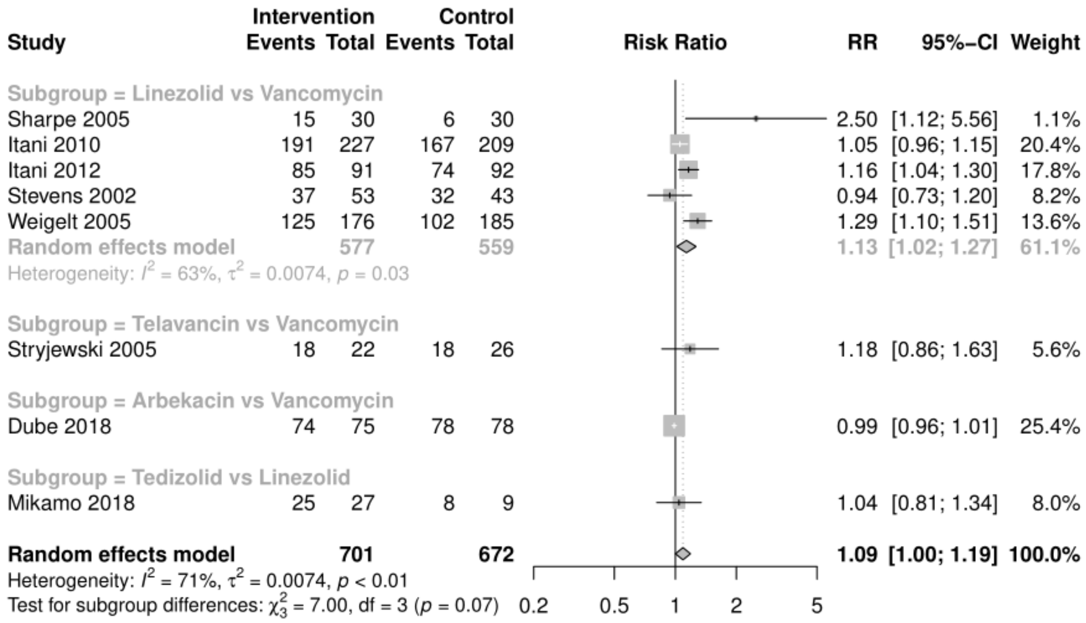

eFigure 7 Direct meta-analysis of clinical cure rates for MRSA cSSTIs

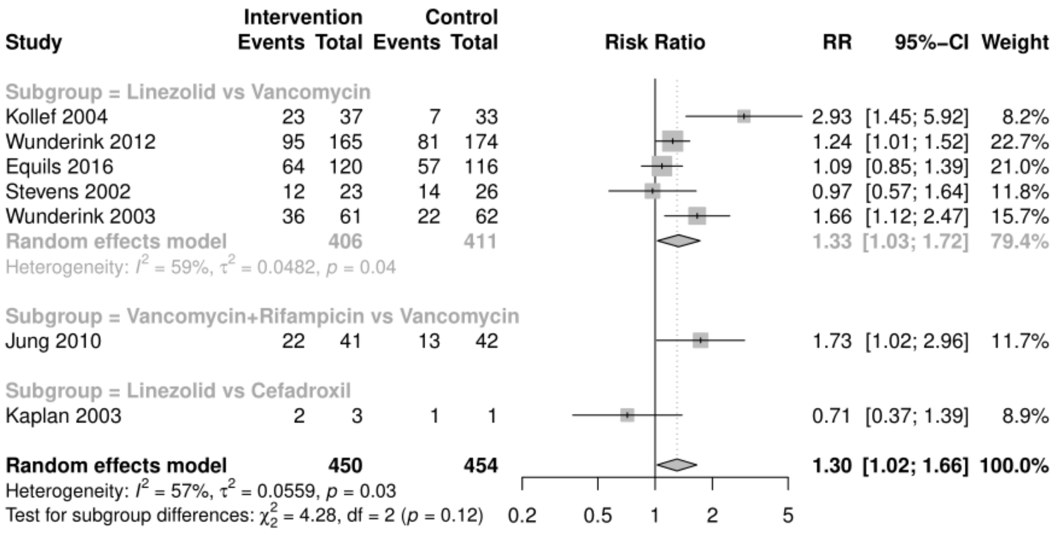

eFigure 8 Direct meta-analysis of clinical cure rates for MRSA pneumonia

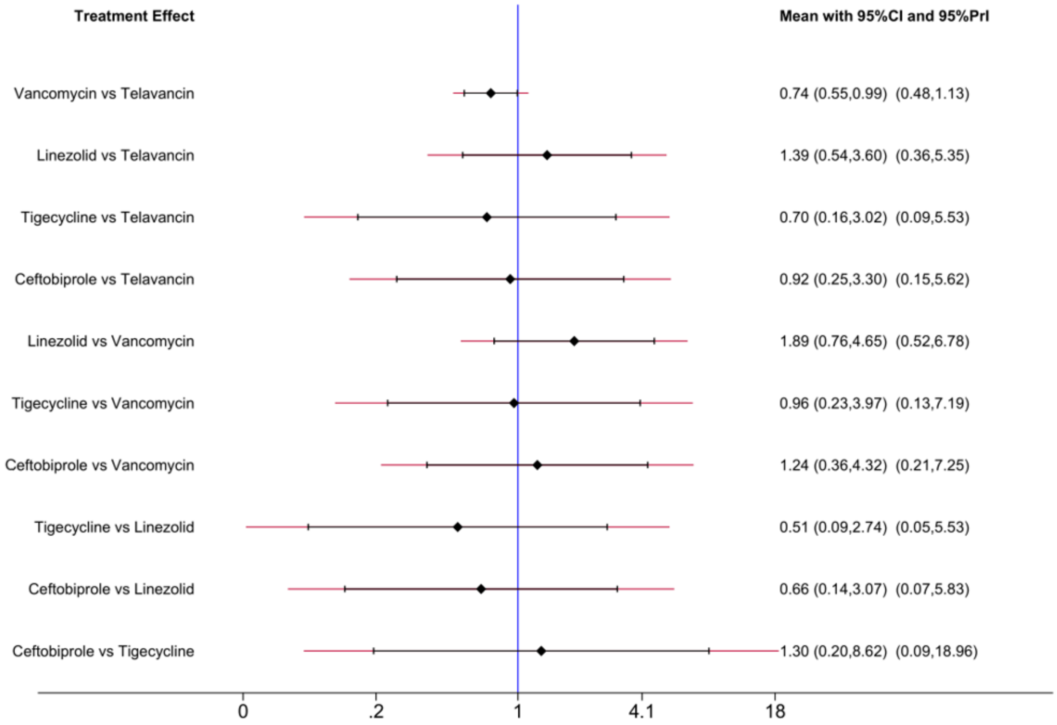

eFigure 9 Network meta-analysis of forest maps of clinical microbiology success rates

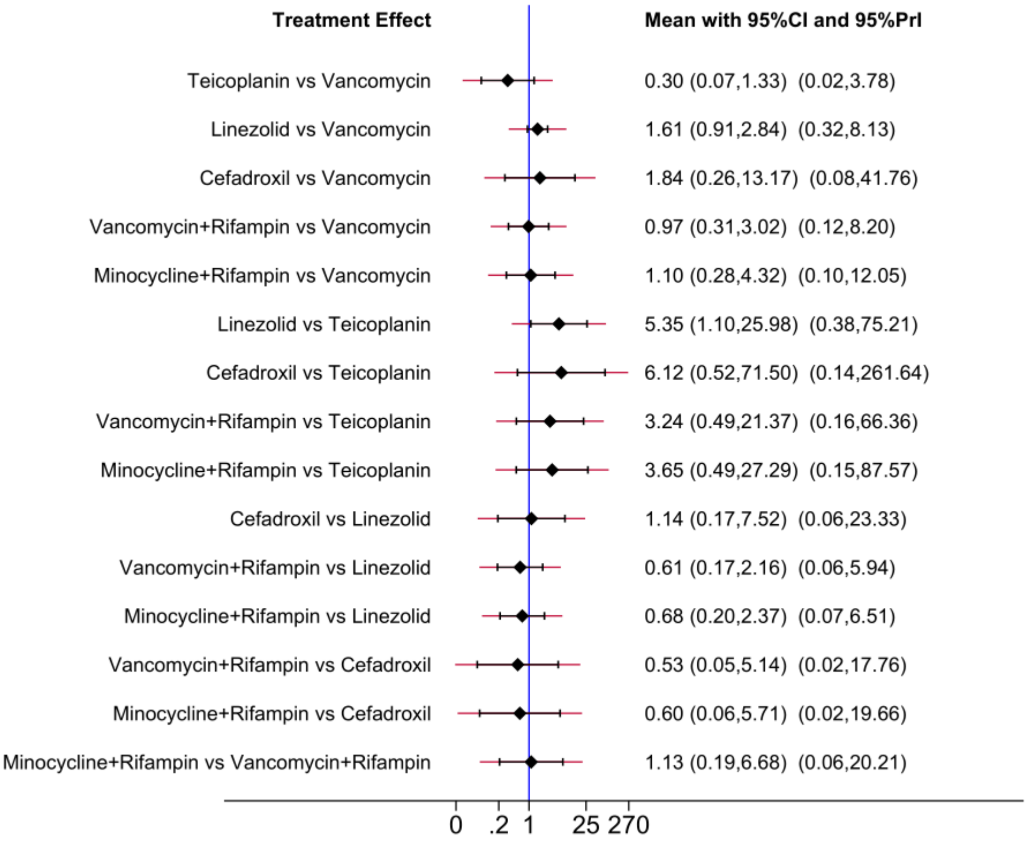

eFigure 10 Network meta-analysis of the incidence of adverse events Forest map

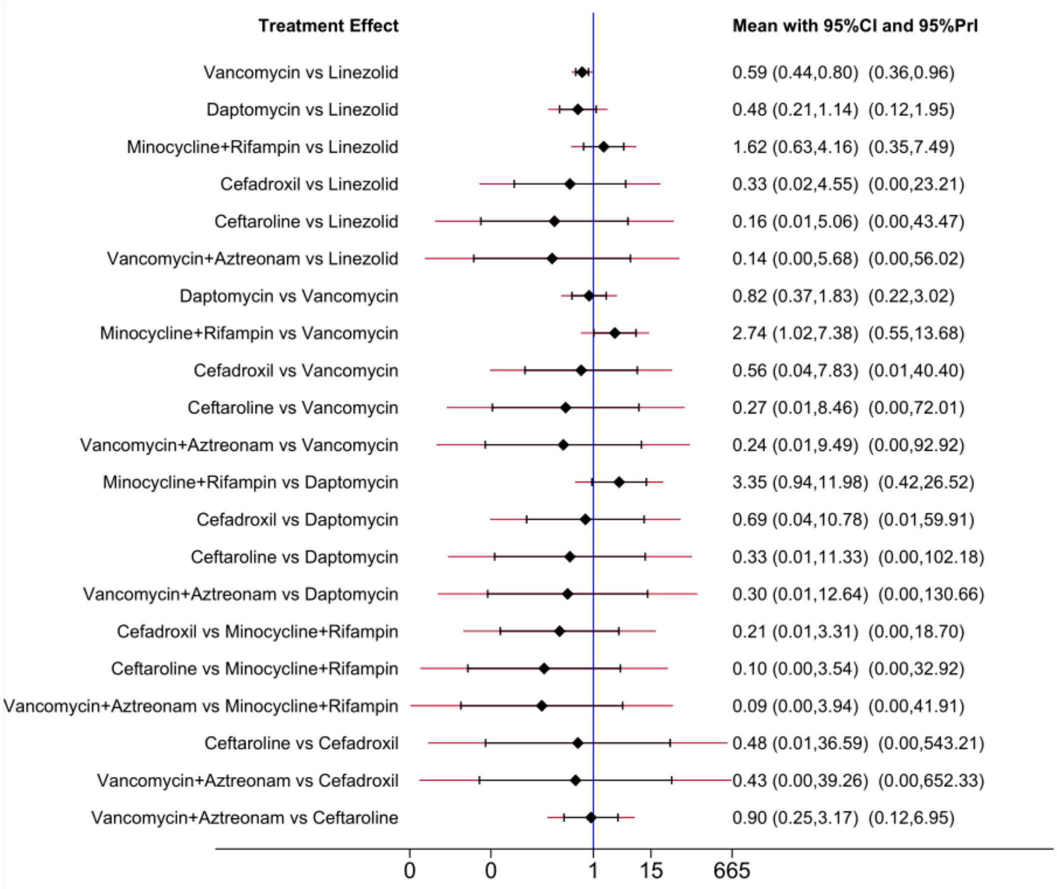

eFigure 11 Network meta-analysis of MRSA cSSSIs clinical cure rate Forest map

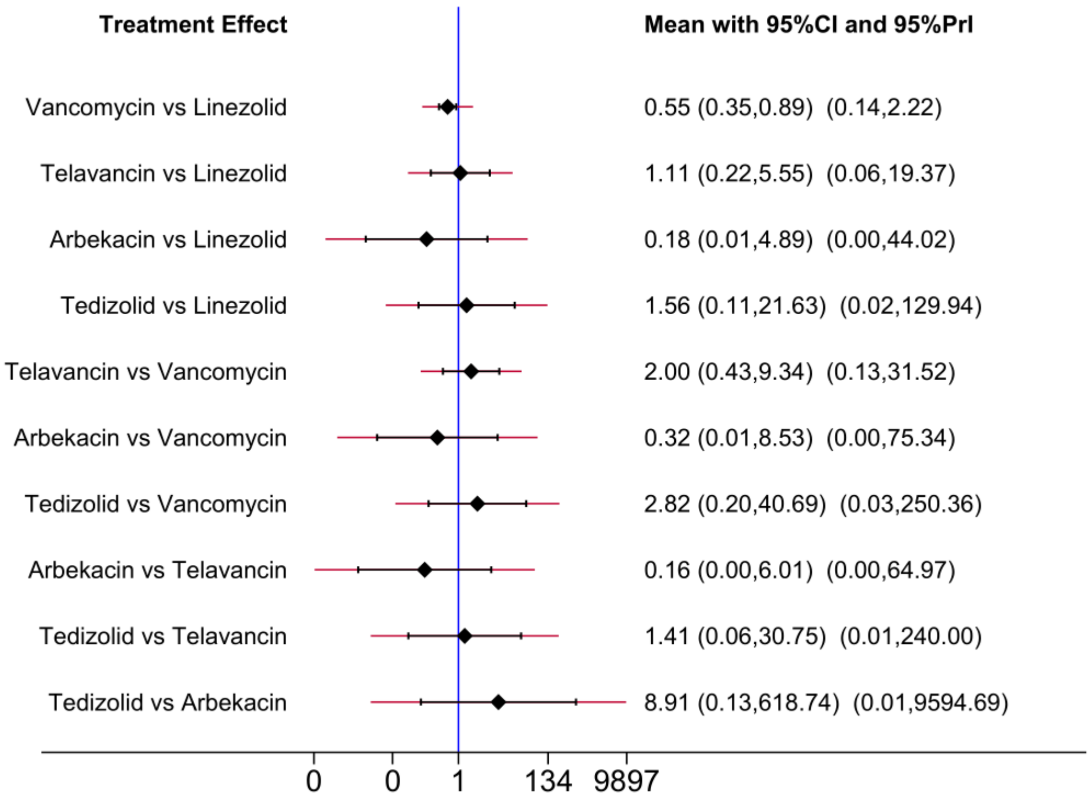

eFigure 12 Network meta-analysis of clinical cure rates for MRSA cSSTIs forest map

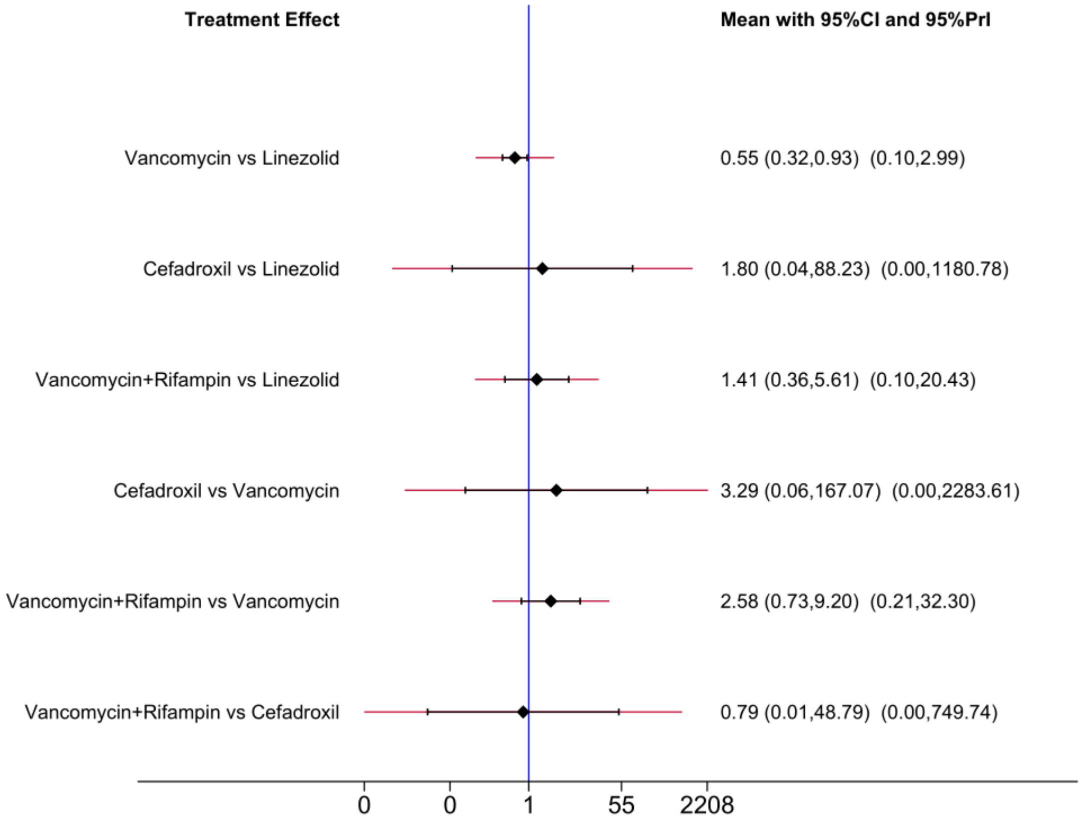

eFigure 13 Network meta-analysis of clinical cure rates for MRSA pneumonia Forest map

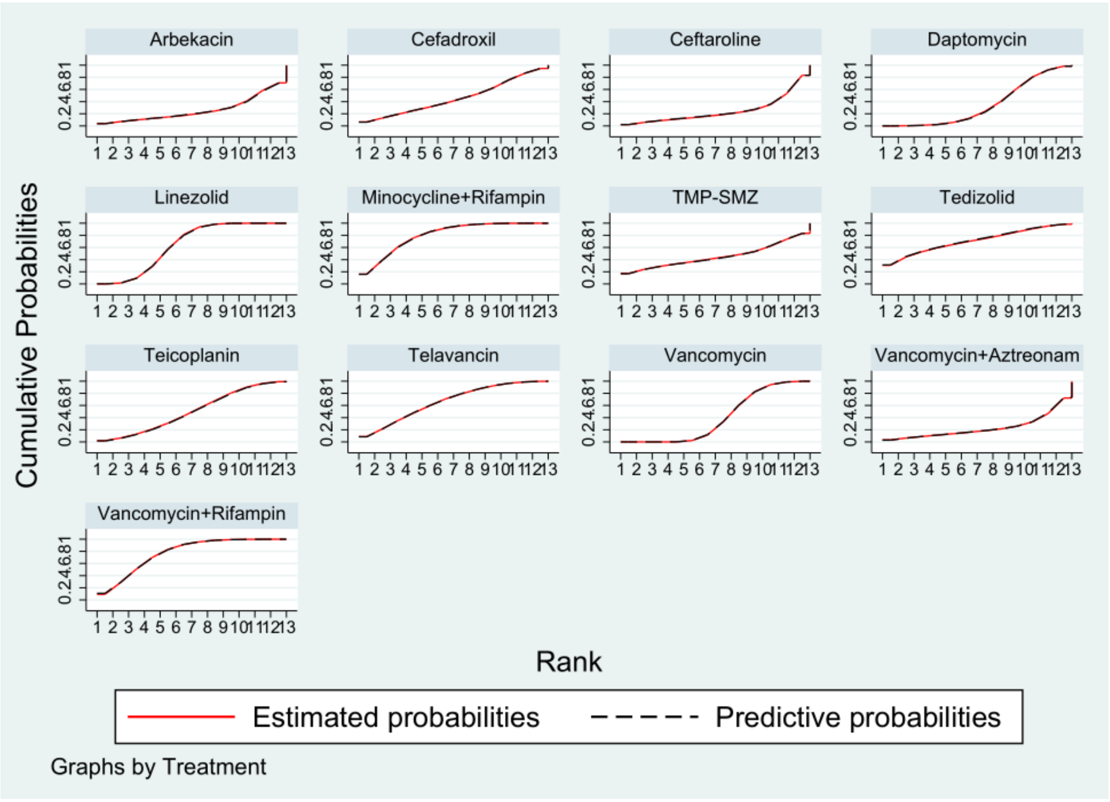

eFigure 14 SUCRA efficacy ranking curve of clinical cure success rate,SUCRA = cumulative ranking surface

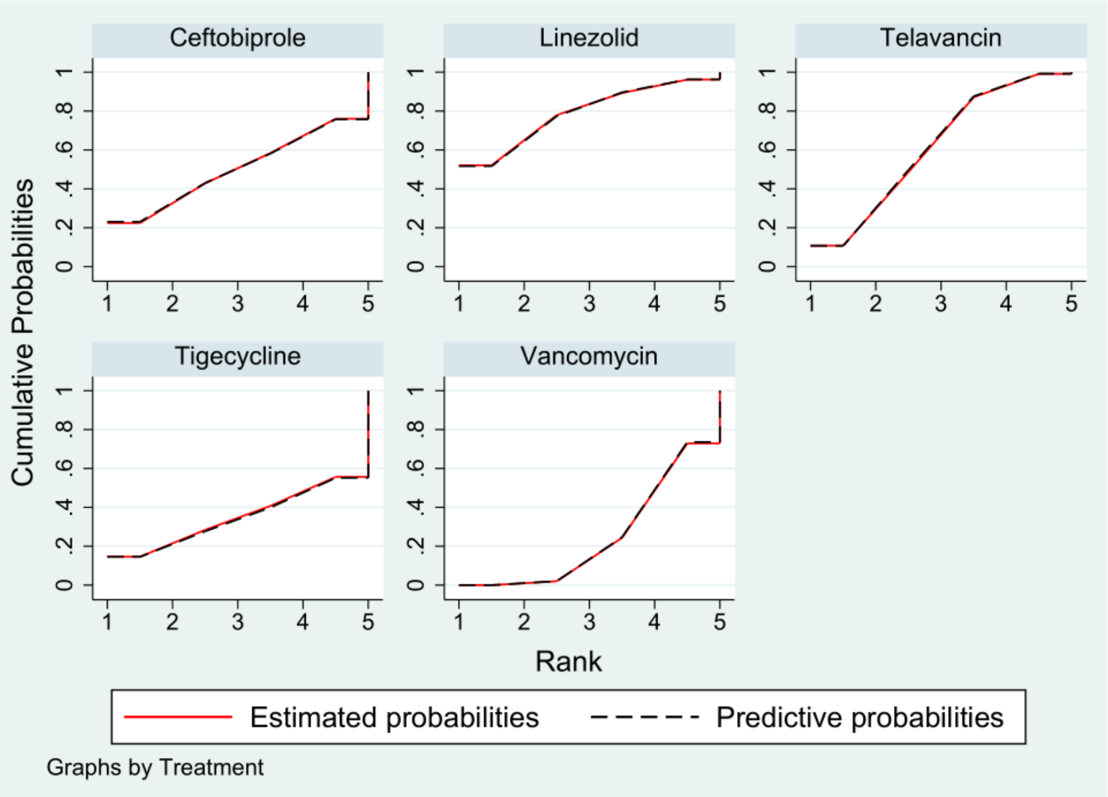

eFigure 15 SUCRA efficacy ranking curve of clinical microbiology success rate, surface under SUCRA = cumulative ranking

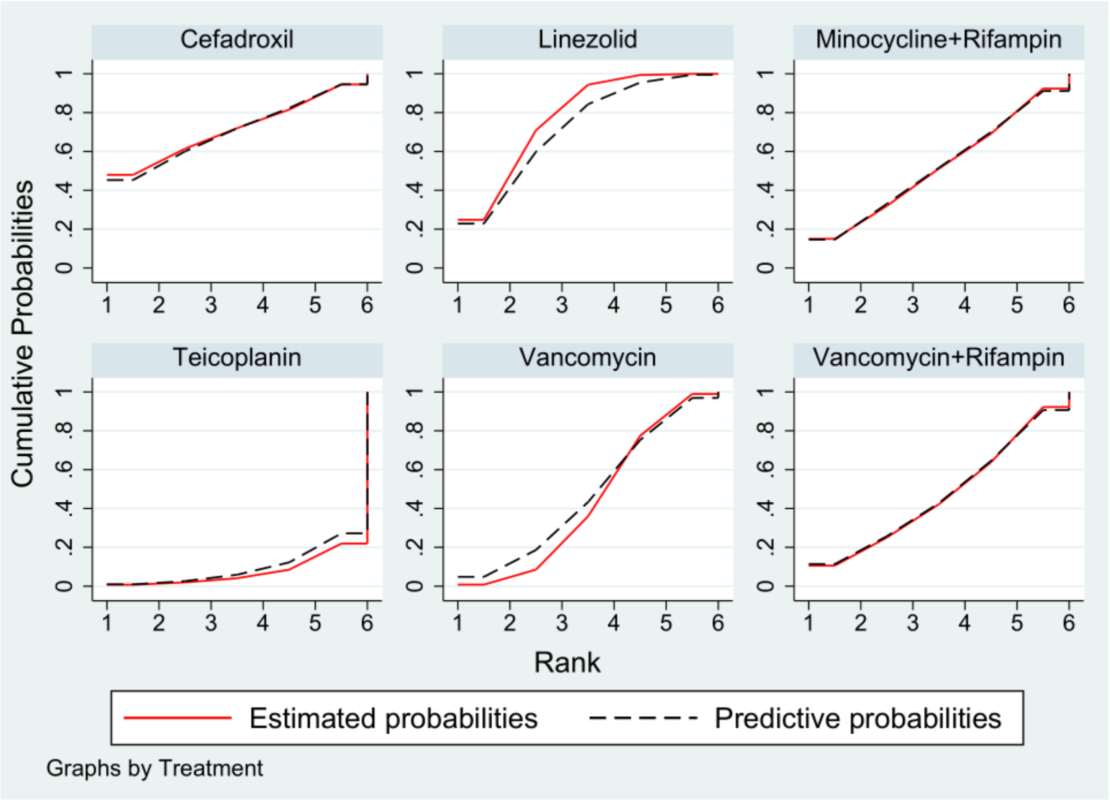

eFigure 16 SUCRA efficacy ranking curve for the incidence of adverse reactions,SUCRA = cumulative ranking under the surface

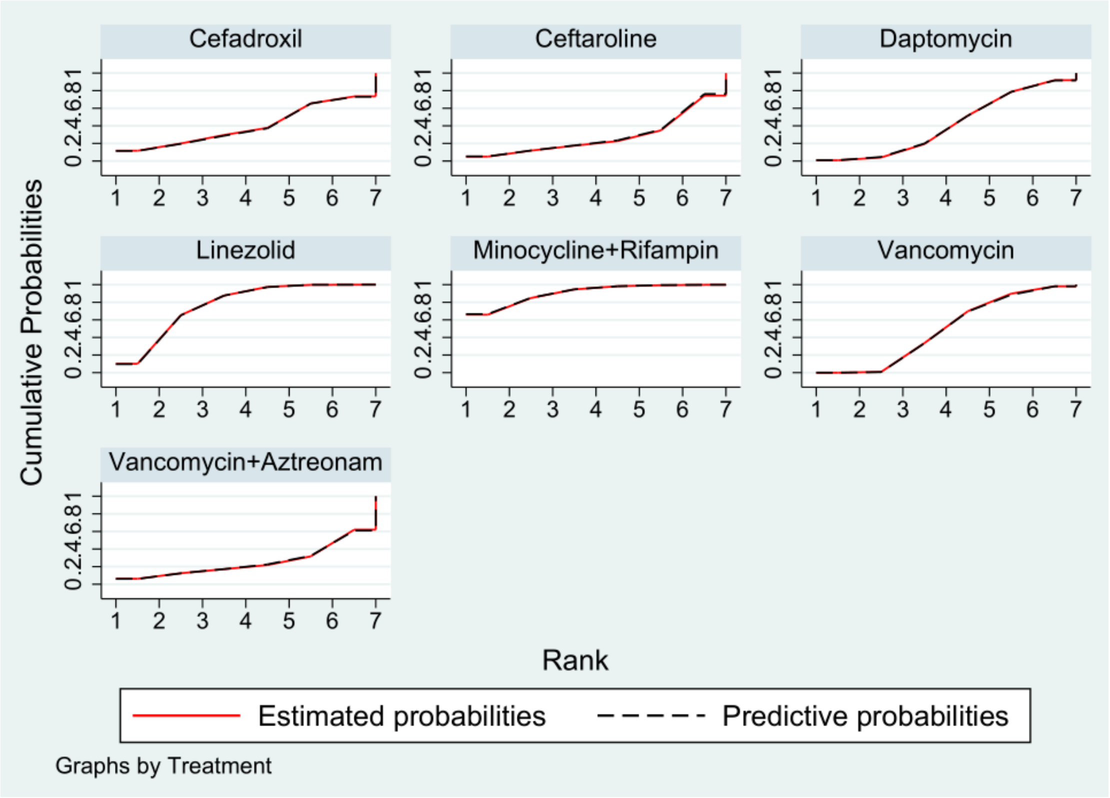

eFigure 17 Clinical cure rate of MRSA cSSSIs SUCRA efficacy ranking curve,SUCRA = cumulative ranking under the surface

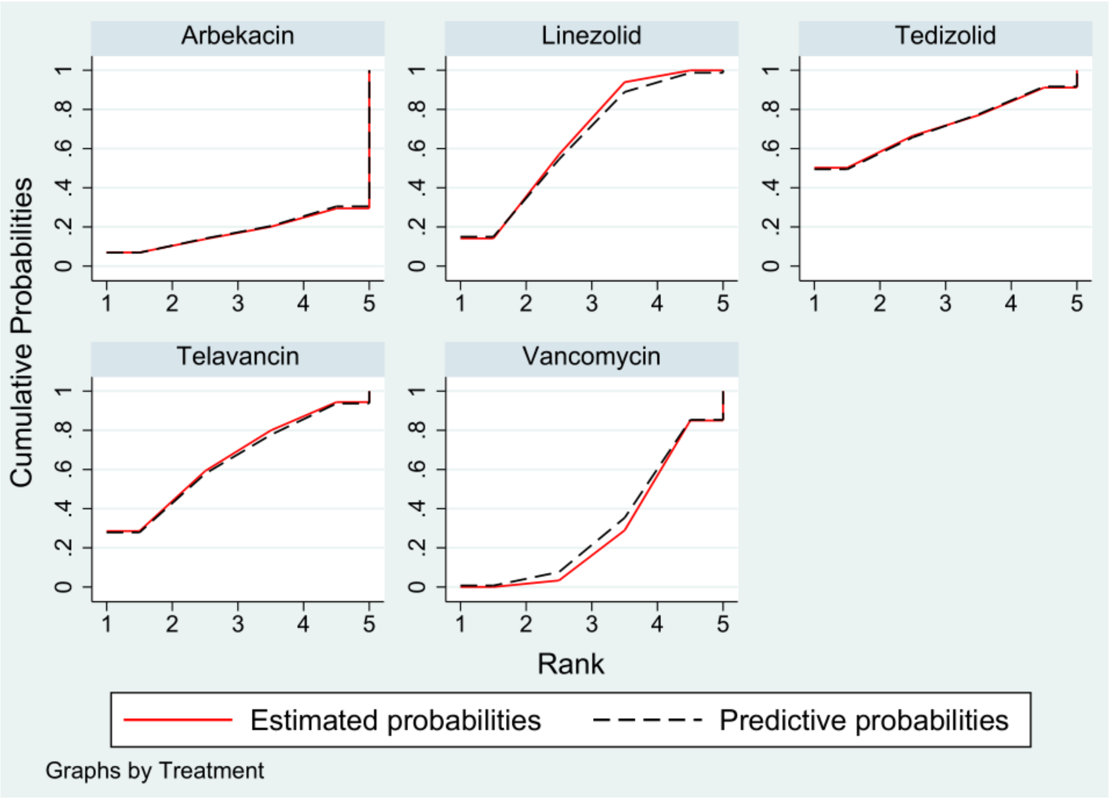

eFigure 18 Clinical cure rate of MRSA cSSTIs SUCRA efficacy ranking curve,SUCRA = cumulative ranking under the surface

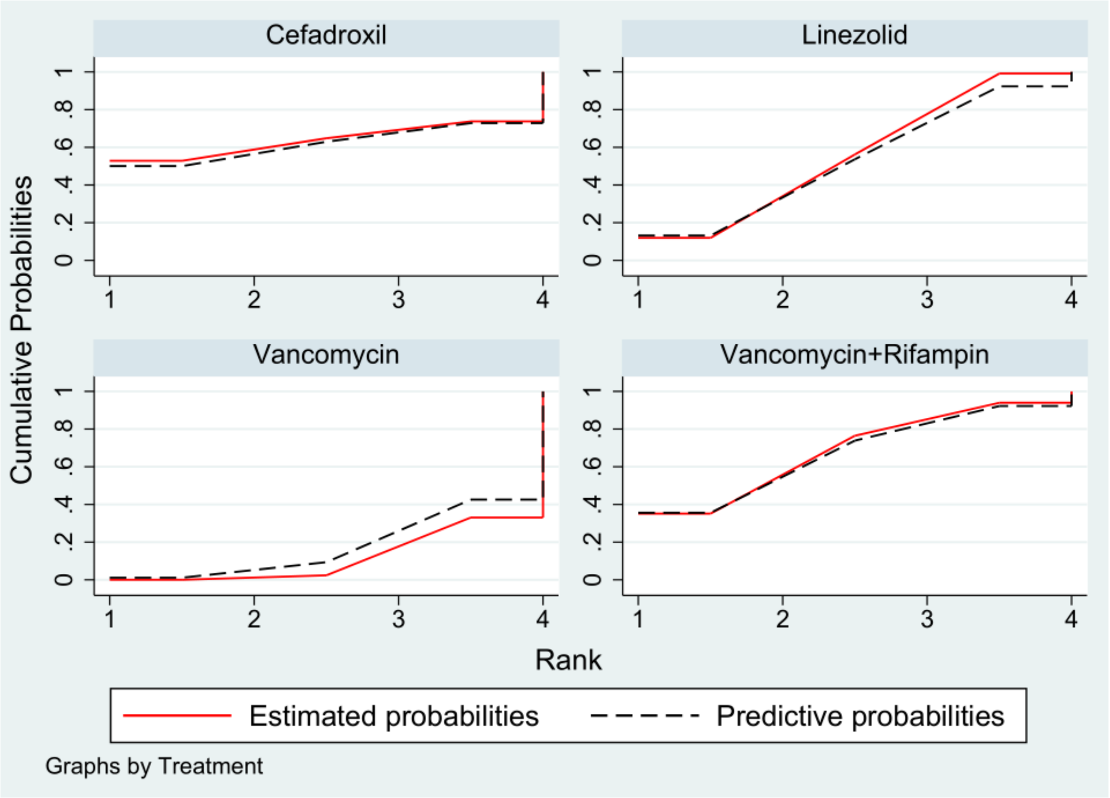

eFigure 19 Clinical cure rate of MRSA pneumonia SUCRA efficacy ranking curve,SUCRA = cumulative ranking surface

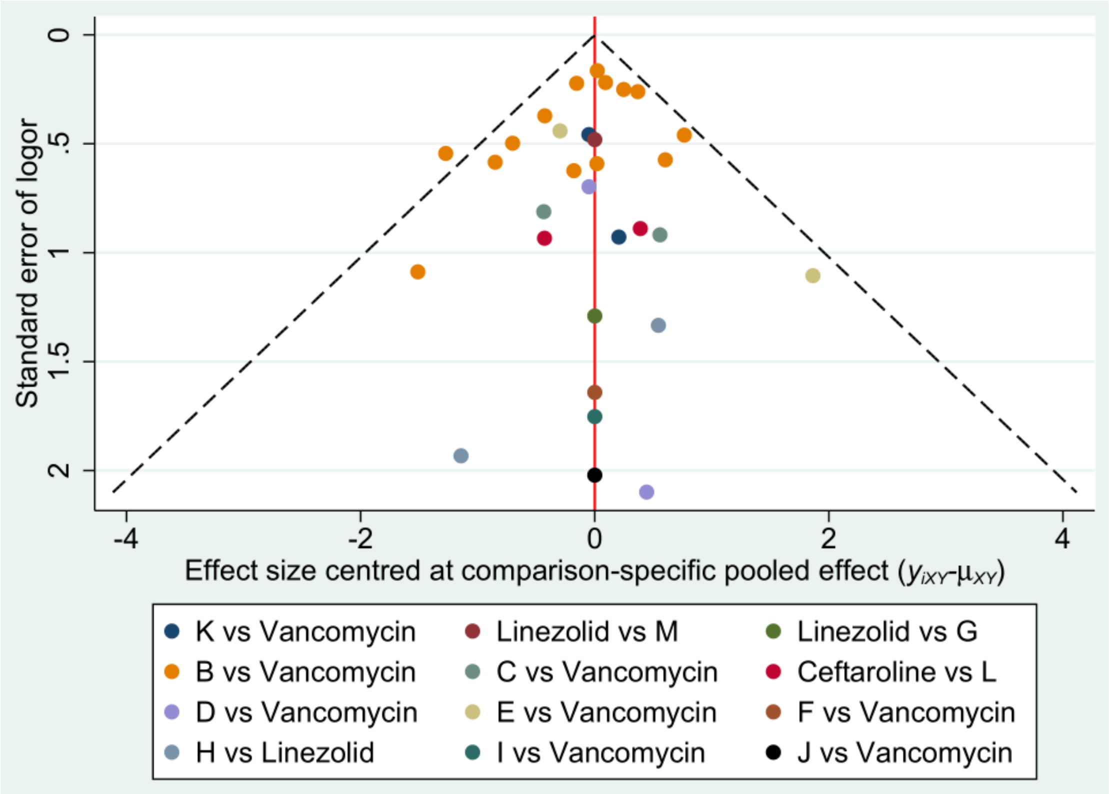

eFigure 20 Funnel plot of a network meta-analysis of clinical cure success rates

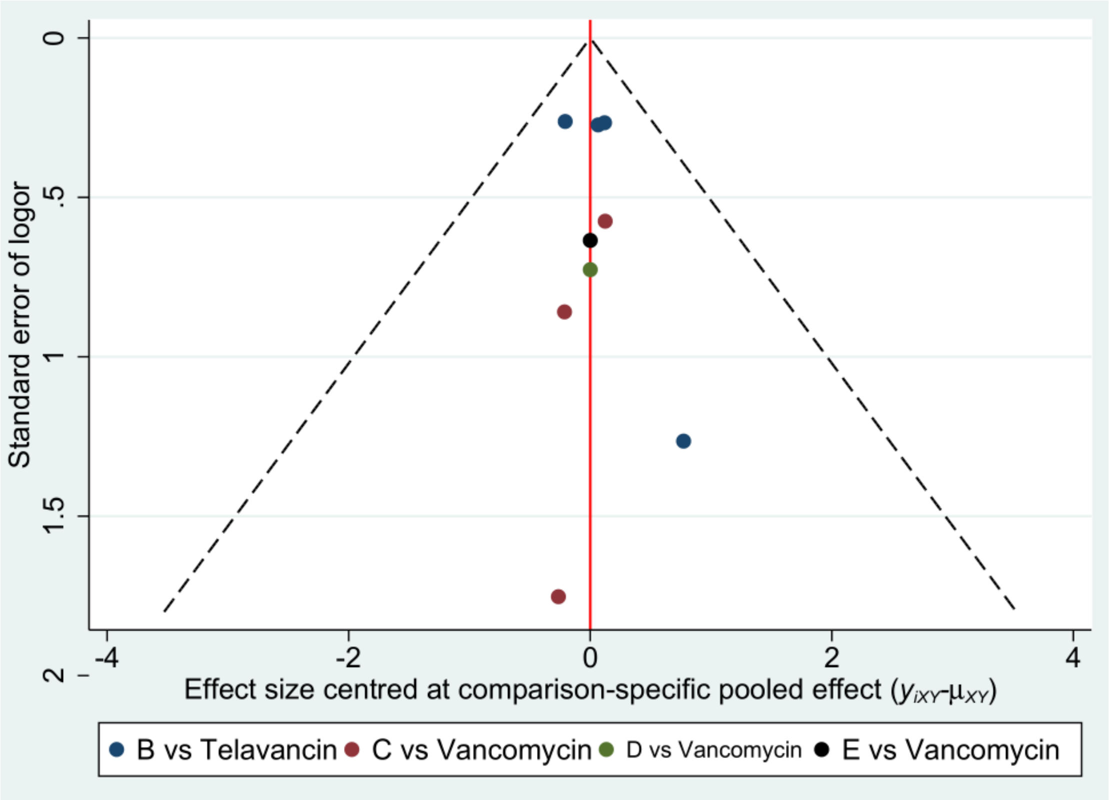

eFigure 21 Funnel plot of a network meta-analysis of clinical microbiology success rates

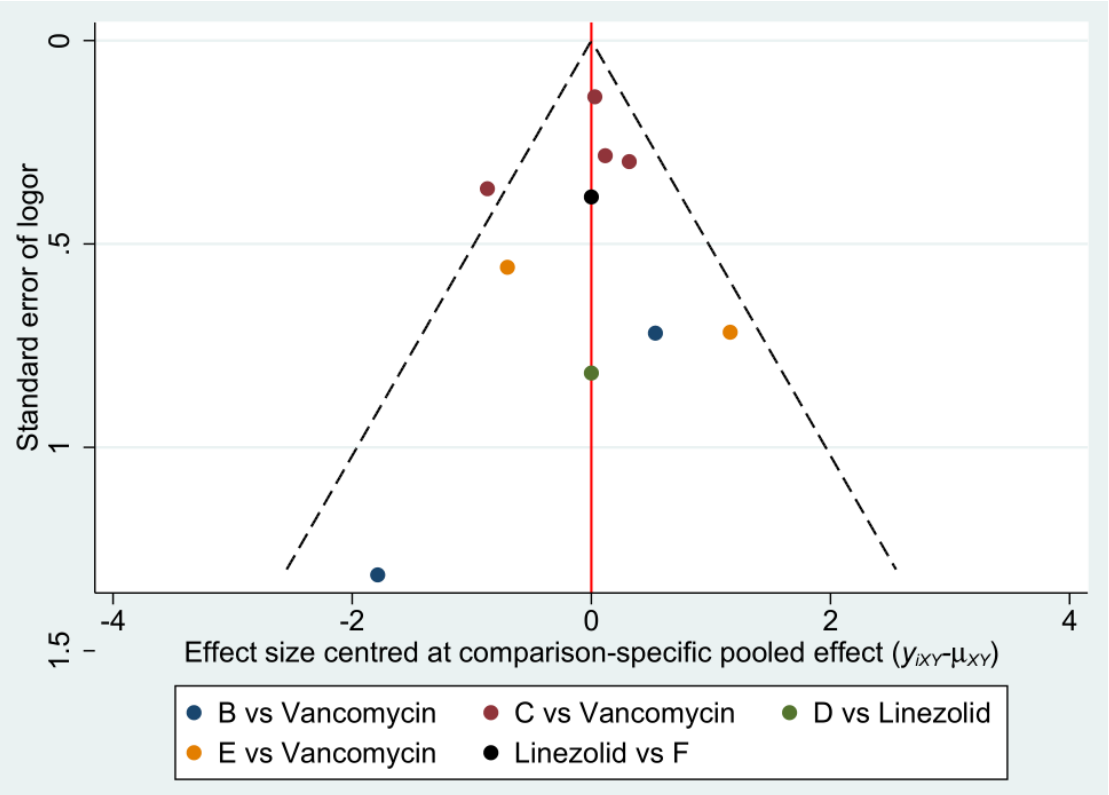

eFigure 22 Funnel plot of a network meta-analysis of the incidence of adverse events

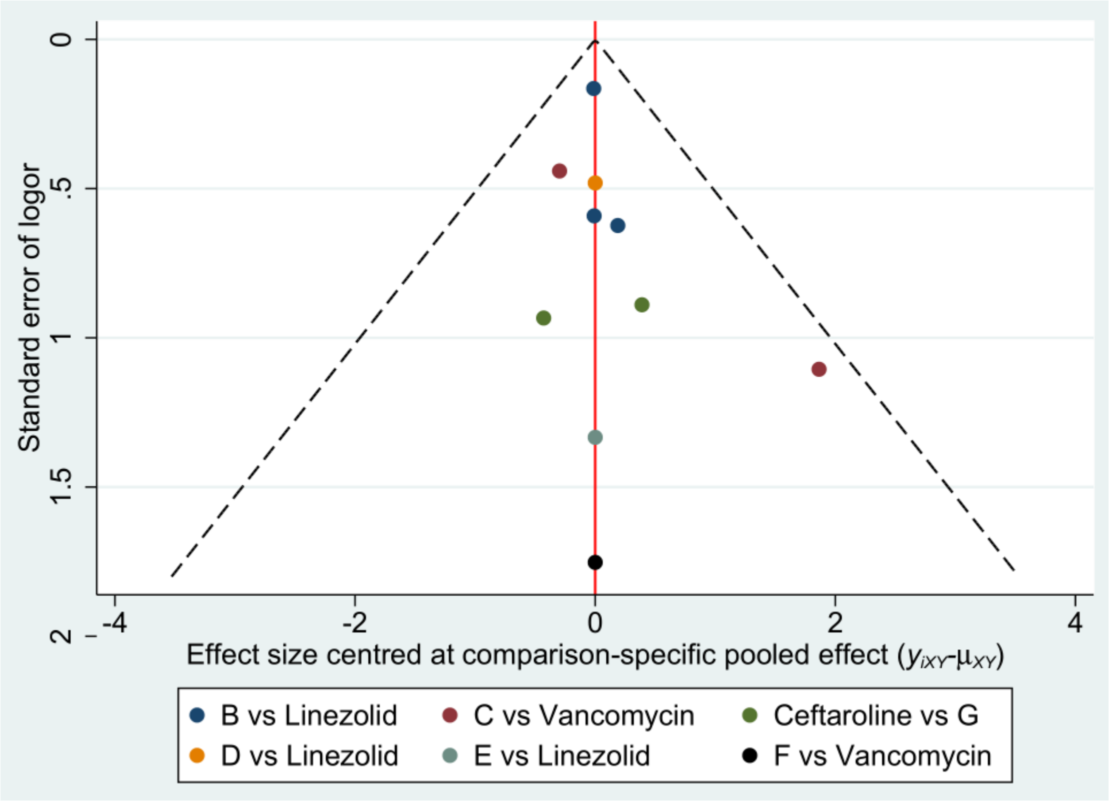

eFigure 23 Funnel plot of a network meta-analysis of clinical cure rates for MRSA cSSSIs

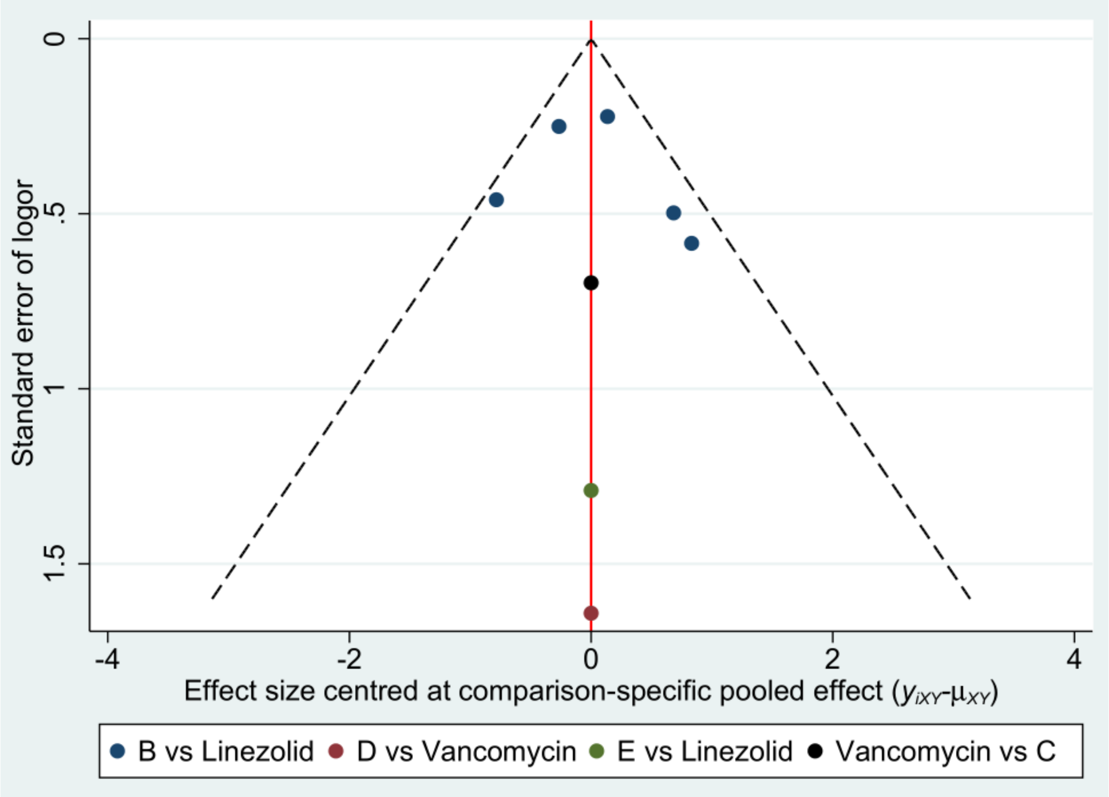

eFigure 24 Funnel plot of a network meta-analysis of clinical cure rates for MRSA cSSTIs

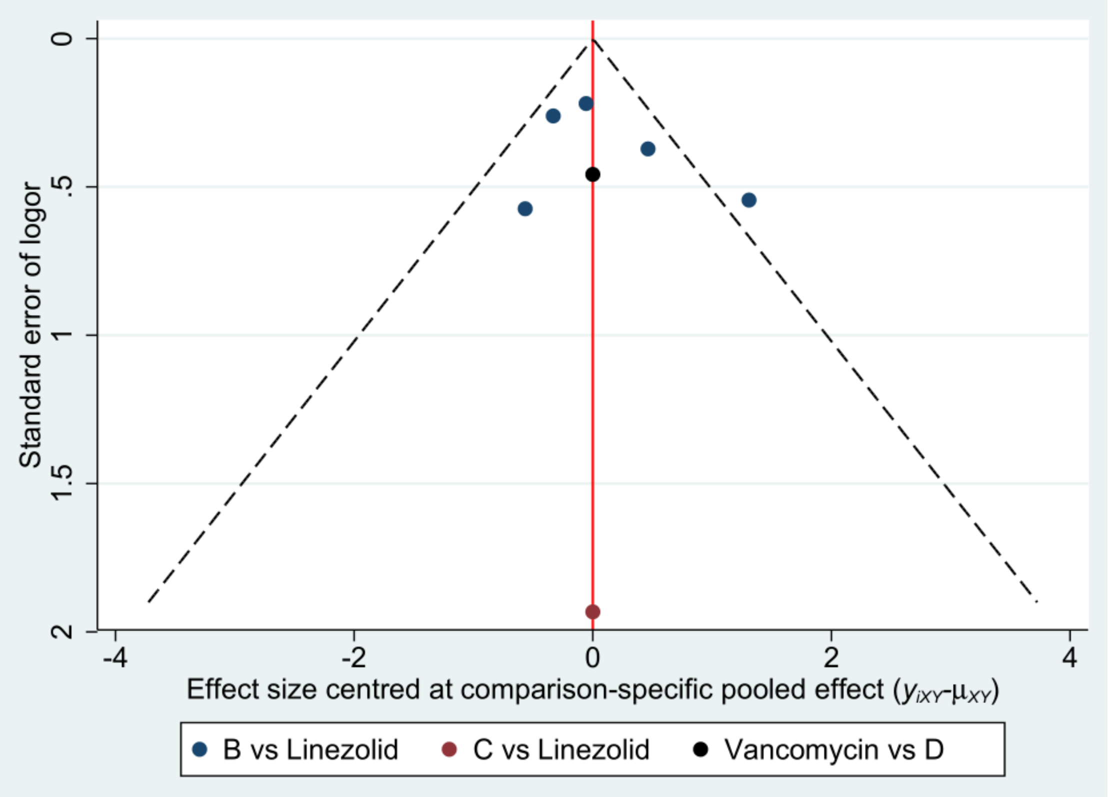

eFigure 25 Funnel plot of a network meta-analysis of clinical cure rates for MRSA pneumonia
